# Association and attributable hospital costs of chronic pain with relevant geriatric sensitive diseases among the older adults

**DOI:** 10.3389/fpubh.2026.1828836

**Published:** 2026-05-01

**Authors:** Ting Chen, Tianjiao Lan, Kun Tan, Jay Pan, Xiuli Wang

**Affiliations:** 1 HEOA Group, West China School of Public Health and West China Fourth Hospital, Sichuan University, Chengdu, China; 2 Institute for Healthy Cities and West China Research Center for Rural Health Development, Sichuan University, Chengdu, China; 3 Sichuan Provincial Maternity and Child Health Care Hospital, Chengdu, China; 4 Health Information Center of Sichuan Province, Chengdu, China; 5 Health Promotion and Food Nutrition & Safety Key Laboratory of Sichuan Province, Chengdu, China

**Keywords:** chronic pain, depression, functional limitation, older adults, avoidable hospital costs

## Abstract

**Background:**

The adverse impacts of chronic pain extend far beyond the physical sensation itself. Chronic pain, an age-related condition, exacerbates geriatric disease burden and drives a central sensitivity-neuropsychiatric complex, necessitating urgent preventive care. This study aimed to investigate the impact of chronic pain on two sensitive diseases, depression, and abilities decline in basic or physical activities (functional limitation) among the inpatients in older adults and explore the attributable hospital costs related to chronic pain.

**Method:**

Participants were sourced from the 2021–2022 Inpatient Discharge Dataset of Sichuan Province, Diagnosis of depression, functional limitation, and chronic pain were identified using International Classification of Diseases, 10th Revision (ICD-10) codes. Logistic regression models were employed to analyze the association between chronic pain and depression and functional limitation. Furthermore, total hospital costs, out-of-pocket costs and length of stay (LOS) were compared between patients (depression and functional limitation) with chronic pain and without using Propensity score matching and Multivariable linear regression.

**Results:**

The analysis included 38,372 and 4,996 inpatients in the depression and functional limitation cohorts, respectively. Chronic pain was significantly associated with both outcomes, yielding odds ratios (ORs) of 1.24 (95% CI: 1.20–1.27) for depression and 1.60 (1.44–1.78) for functional limitation (all *p* < 0.001), and the effect intensified as the number of painful areas increased. Compared to those without pain, depression patients with chronic pain incurred 68.2% higher total hospital costs ( β =0.52, *p* < 0.001), 169.1% higher out-of-pocket ( β =0.99, *p* < 0.001) and 60.0% higher LOS ( β =0.47, *p* < 0.001). Among patients with Functional limitation, chronic pain also significantly increased log-transformed total costs ( β =0.20), out-of-pocket ( β =0.51), and LOS ( β =0.30), representing relative increases of 22.1, 66.5, and 35.0%, respectively (all *p* < 0.05). These economic impacts were more pronounced among patients with multi-area pain.

**Discussion:**

This study provides empirical evidence linking chronic pain to deteriorated psychological and physical health among older adults. It highlights the increased burden of the disease and hospitalization, with a particular emphasis on the dangers of multi-area pain. These findings emphasize that prioritizing mental health-focused pain management in outpatient and emergency settings is crucial for preventing avoidable hospitalizations and hospitalization costs in older adults.

## 1 Introduction

As an age-associated condition, chronic pain has become one of the primary complaints among older adults, impacting their quality of life (1). Pain is formally defined as an unpleasant sensory and emotional experience associated with, or resembling that associated with, actual or potential tissue damage (2). Beyond localized pathology, chronic pain frequently coexists with various geriatric syndromes, such as physical dysfunction, falls, disability, and persistent depression (3, 4, 5). Serving as a potent physical and psychological stressor (3, 4), chronic pain can trigger or exacerbate psychological distress and mobility limitations (6). Consequently, there is an urgent need to prioritize preventive measures and intervention strategies within primary and ambulatory care settings to mitigate its multi-dimensional impact (2, 7–9).

The epidemiological and economic burdens of chronic pain are substantial. The pooled prevalence of persistent pain is estimated at 34% in lower- and middle-income countries (10), while recent data indicate that approximately 20.9% of U. S. adults suffer from chronic pain (11), and this prevalence increases with age, ranging from 27 to 86% in older demographics (11, 12). Notably, this burden is exacerbated by central sensitization, a state of spinal cord hyperexcitability, and neuropsychiatric alterations, which create a pathophysiological bridge linking chronic pain with depression and functional decline (13, 14). Specifically, persistent pain can induce neuroplastic changes in brain regions governing mood and motor control, thereby precipitating or worsening depressive symptoms and mobility limitations (15, 16). Economically, chronic pain imposes a significant economic burden positively associated with age (14, 17, 18). For instance, researchers estimate that chronic pain results in additional healthcare costs of $261–$300 billion (in 2010 dollars) annually in the United States (19).

Beyond the direct economic burden caused by these chronic pains, chronic pain frequently coexists with various geriatric syndromes such as physical dysfunction, falls, and disabilities (5). Moreover, pain serves as a significant physical and psychological stressor capable of triggering or exacerbating psychological distress (6). With aging, there is a steady increase in the prevalence of depression and functional limitation among older adults (20), and chronic pain potentially exacerbating the onset and progression of these conditions. In China, the rapid aging of the population has amplified the severity and urgency of managing chronic pain and its co-occurring syndromes (21). Moreover, awareness regarding pain management among Chinese residents remains alarmingly low. A national survey revealed that 24.06% of respondents did not seek medical help, and 36.79% never received any treatment because they believed chronic pain is not harmful (22). While several epidemiological and clinical studies have independently explored the associations between chronic pain and depression, or functional limitation (15, 23–25). Despite offering partial insight into the effects of chronic pain, significant knowledge gaps persist. Specifically, evidence derived from large-scale hospitalization data remains notably limited (26). Furthermore, the additional medical burden driven by chronic pain warrants special attention, particularly among patients with depression and functional limitation. Because these syndromes often manifest as non-emergency conditions, effective early intervention and pain management could potentially prevent disease exacerbation and subsequent costly hospitalizations.

Drawing on data from the Inpatient Discharge Dataset of Sichuan Province, this study aimed to: (1) explore the associations of chronic pain with depression and functional limitation among older adults; and (2) assess the attributable hospital costs associated with chronic pain within these specific disease patients.

## 2 Methods

### 2.1 Study design and participants

The Inpatient Discharge Dataset of Sichuan Province, sourced from the Health Commission of Sichuan Province (2021–2022) was used to investigate the association of chronic pain with depression and functional limitation, as well as its attributable hospital costs. Sichuan Province has a population of about 83.7 million, accounting for roughly 6% of the national population, and registers an average of over 17 million hospital admissions annually. Covering an area of approximately 485,000 square kilometers, Sichuan comprises of 21 cities or prefectures and 183 counties (as of 2020). Notable, the province is intersected by the Hu’s Line, a major geo-demographic demarcation line separating China based on population density, geography, and economic development. Consequently, Sichuan is widely regarded as a representative microcosm of China’s broader demographic and epidemiological landscape (27).

The Inpatient Discharge Dataset encompasses comprehensive records of all hospitalizations during the observation period, including information on inpatients’ basic characteristics, as well as diagnostic and treatment data. We extracted patient-level data on demographics (including age, gender, residence type and insurance health program, permanent address, ethnicity and marital status); admission time; the names of the principal and secondary diagnoses, as well as their codes from the International Classification of Diseases, 10th Revision (ICD-10); discharge time; patient identity (patient unique identifiers were encrypted to safeguard privacy) and detailed medical expense information. Consistent with the inclusion and exclusion criteria for the main analysis, patients aged < 45 years and with missing key variables were excluded from this analysis. Furthermore, to rigorously handle missing data, we employed a complete-case analysis approach; individuals with missing key variables were excluded from the final analytical cohort. The detailed step-by-step inclusion and exclusion process is illustrated in Supplementary Figure S1.

To align with the chronic nature of the conditions under study, we transformed the longitudinal hospital records into an individual-level cross-sectional dataset to accurately reflect cumulative health impacts. The analytical processing of key variables was structured as follows: (1) disease Identification: a diagnosis of diseases or chronic pains was assigned if an individuals had related hospitalizations during the 2021–2022 study period; (2) economic Outcomes: hospital utilization metrics, specifically total hospital costs, out-of-pocket costs, and length of stay (LOS), were calculated by aggregating all inpatient episodes for each individual and converting them into annualized averages; (3) covariates: individual demographic and baseline characteristics were derived from the 2021 enrollment year to ensure temporal consistency within the regression models. Ultimately, the final datasets included 13,148,997 participants, and in which 38,372 and 4,996 for depression and functional limitation patients, respectively.

### 2.2 Assessment of chronic pain and outcomes

Because the Inpatient Discharge Dataset relies on standardized hospitalization records, International Classification of Diseases, 10th Revision (ICD-10) codes were utilized to identify all relevant conditions. The specific ICD-10 codes for the outcomes, principal chronic pain, and chronic secondary pain syndromes across different anatomical sites were determined based on previous studies and the standardized ICD coding system utilized in China (28). A comprehensive list of the diagnostic codes for the various chronic pain sites and study outcomes is provided in Supplementary Table S1, and their corresponding frequency distributions within the dataset are summarized in Supplementary Table S2.

Furthermore, because direct clinical assessments of basic physical activities of daily living (ADLs) could not be fully captured within this administrative dataset, we utilized specific diagnostic conditions as proxy variables. This selection process was rigorously guided by existing literature and expert clinical consultations (29–31). Specifically, functional limitation was operationalized using a cluster of relevant diagnostic codes, including abnormal gait and mobility (R26), dyspnea on exertion (R29.3), and issues related to care-dependency and ADLs (Z73).

### 2.3 Calculation of costs

For each inpatient, comprehensive cost data for the entire hospital stay were extracted from the medical records. Three primary economic outcomes were evaluated in this analysis. First, total hospital costs were calculated as the sum of expenses for all medical service, treatment operations, nursing service and other costs in hospitalization. Second, out-of-pocket costs were estimated as the remaining total hospital cost after reimbursement from the basic medical insurance system in China. Third, as a measure of time cost, we also estimated the length of hospital stay (LOS) as a proxy variable for opportunity cost. Finally, to standardize these metrics, all relevant indicators were derived by pooling all inpatient episodes at the individual level and subsequently normalizing them into annualized averages.

### 2.4 Statistical analysis

The baseline characteristics of all participants, stratified by the presence of chronic pain, were summarized using means ± standard deviation (*Mean* ± *SD*) for continuous variables or *n* (%) for categorical variables. To comprehensively explore the associations of chronic pain with depression and functional limitation, as well as to estimate the attributable hospital costs, our statistical analysis was conducted in two distinct analytical steps.

Step 1: Logistic regression models within a generalized linear model framework based on the cross-sectional design were used to assess the association (odds ratio, OR) of chronic pain with depression and functional limitation using the dataset of all participants. Separate analyses were conducted using the presence for each specific condition (depression or functional limitation) as the primary outcome. For each disease, two distinct models were constructed after controlling for all covariates. First, in the Model 1 evaluated the binary impact of chronic pain by dividing patients into two groups: those with and those without chronic pain. Model 2 further categorized patients based on the number of chronic pain areas (1 area, 2 areas, or ≥ 3 areas) to evaluate the cumulative impact of multi-area pain. All models were adjusted for a comprehensive set of covariates, specifically: age, sex, regional economic level, ethnic group, marital status, occupation type (categorized as predominantly physical labor versus mental labor), and health insurance type. Detailed operational definitions of these covariates are provided in Supplementary material S1.

Step 2: To test for an association of chronic pain with hospital costs, we used propensity score matching and multivariable linear regression to predict total costs, out-of-pocket and LOS, controlling for covariates. Specifically, the covariates included in the propensity score model were age, sex, regional economic level (high, medium and low), ethnic group (minority and non-minority), marital status (Married and Unmarried/divorced/widowed), occupation type (Mental worker, Manual workers and Uncertain), and health insurance type (Employee medical insurance, Resident medical insurance and Others). Patients were matched using a 1:1 nearest-neighbor approach without replacement, with a caliper set to 0.2 standard deviations of the logit of the propensity score.

To examine the specific impact of chronic pain on hospital costs, the dataset was stratified by diseases, with separate models estimated for patients with depression and those with functional limitations. Because the cost models were estimated within disease-specific groups (e.g., all patients having depression), the baseline costs of the underlying disease are inherently controlled for, allowing the models to isolate the additional costs attributable to chronic pain. Prior to regression modeling, we examined the cost distribution. There were no issues with negative costs or missing value identified, and the log-transformed of total hospital costs, out-of-pocket and LOS approximated normal distribution (The result of residuals tests are presented in Supplementary Figure S2). We then used *β* coefficients to estimate mean adjusted log-cost change for patients with chronic pain. Given that some economic outcome variables contained zero values, all dependent variables (hospital costs and LOS) were log-transformed using the ln(y+1) function to approximate a normal distribution and ensure model stability. The resulting regression β coefficients were subsequently interpreted as relative percentage changes using the formula (exp(β)−1) times 100%. Furthermore, the economic impact of multi-area chronic pains was additionally explored.

To validate our findings, a series of robustness checks were conducted. First, we re-estimated the models using the unmatched data. Second, we performed regressions on the raw, unlogged cost variables to provide directly interpretable results on an absolute scale.

Data management and all analyses were carried out using R 4.3.1 software. Two-sided *p* < 0.05 were considered statistically significant.

## 3 Results

### 3.1 General characteristics and costs

The baseline characteristics and hospital costs of the participants, stratified by the presence of chronic pain, are presented in Table 1.

**Table 1:** The participants’ baseline characteristics according to outcomes.

Variables	Depression		*p*	Functional limitation		*p*
	With pain	Without pain		With pain	Without pain	
*N*	5,454	32,918		407	2,091	
Age, Mean (SD)	65.53 (11.31)	63.36 (11.54)	<0.001^***^	67.52 (11.81)	67.70 (11.76)	0.331
Female (ref = Male)	74.37 (73.19, 75.51)	68.27 (67.76, 68.77)	<0.001^***^	45.7 (40.92, 50.56)	39.84 (37.76, 41.95)	0.035
Regional economic level (ref = Medium)			<0.001^***^			0.215
High	64.8 (63.52, 66.05)	62.18 (61.65, 62.70)		78.62 (74.38, 82.33)	78.43 (76.62, 80.14)	
Low	16.26 (15.31, 17.27)	18.45 (18.03, 18.87)		10.32 (7.73, 13.66)	12.48 (11.13, 13.97)	
Non-minority ethnic group (ref = Minority)	97.21 (96.74, 97.62)	97.72 (97.55, 97.87)	0.026^*^	98.77 (97.16, 99.47)	98.95 (98.41, 99.30)	0.827
Married (ref = Unmarried, divorced or widowed)	85.3 (84.33, 86.21)	85.57 (85.19, 85.95)	0.603	79.85 (75.68, 83.46)	85.37 (83.79, 86.82)	0.245
Occupation type (ref = Uncertain)			<0.001^***^			0.146
Mental worker	4.33 (3.82, 4.90)	6.00 (5.75, 6.26)		3.93 (2.43, 6.29)	2.73 (2.11, 3.52)	
Manual workers	34.62 (33.37, 35.89)	33.47 (32.96, 33.98)		36.36 (31.84, 41.14)	31.47 (29.51, 33.49)	
Health insurance type (ref = Others)			<0.001^***^			0.188
Employee medical insurance	37.42 (36.15, 38.71)	32.86 (32.36, 33.37)		32.92 (28.54, 37.63)	31.9 (29.94, 33.93)	
Resident medical insurance	38.28 (37.00, 39.58)	41.06 (40.53, 41.59)		42.51 (37.80, 47.36)	38.55 (36.48, 40.65)	
Annually total hospital costs per patient, Median (IQR), $	2,118.04 (1,129.89, 4,035.96)	1,174.82 (611.45, 2,415.57)	0.218	3,211.39 (1,371.76, 7,370.85)	2,619.88 (1,070.04, 6,252.14)	0.109
Annually out-of-pocket hospital costs per patient, Median (IQR), $	337.56 (56.06, 956.43)	184.61 (0, 592.66)	0.253	337.55 (56.05, 956.42)	163.40 (0, 681.54)	0.239
Annually Length of hospital stay per patient, Median (IQR), days	21.00 (11.50, 36.50)	11.00 (6.00, 21.51)	0.231	26.50 (13.00, 49.88)	20.50 (10.01, 43.03)	0.002^**^
No. of pain areas						
1	82.8 (81.78, 83.78)			80.84 (76.73, 84.36)		
2	14.81 (13.90, 15.78)			16.46 (13.18, 20.38)		
>2	2.38 (2.01, 2.82)			2.70 (1.52, 4.77)		

The details of covariates shows in Supplementary material S1; Continuous variables (Age) are presented as mean (SD), SD means Standard Deviation; and Discrete variables are presented as *n*% (confidence interval); Costs are presented as Median (IQR), IQR means Interquartile Range; ref means the reference group; The costs are adjusted to the amount in US dollars for the year 2021. *** *p* < 0.001, ** *p* < 0.01, * *p* < 0.05, Differences between groups were evaluated using the student’s t-test for normally distributed continuous variables, the Mann–Whitney U test for skewed continuous variables, and the Chi-square test for categorical variables.

A total of 38,372 and 4,996 participants aged 45 years and older were included in the depression and functional limitation analyses datasets, respectively. Overall, a higher proportion of females was observed among participants experiencing chronic pain. Specifically, within the depression cohort, patients with chronic pain were slightly older than those without pain (mean age: 65.53 and 63.36 years, respectively). Additionally, a significantly higher proportion of females was observed in the pain group compared to the non-pain group (74.37 and 68.27%). Similarly, in the functional limitation cohort, the chronic pain group contained a higher percentage of females (45.70 and 39.84%), although the mean age was comparable between the two subgroups (67.52 and 67.70 years). Across all cohorts, the majority of participants resided in regions with high economic development. In terms of occupation, individuals engaged in physical labor constituted a larger proportion of the population than those in mental labor across all groups. Regarding health insurance coverage, resident medical insurance and employee medical insurance were the predominant types, accounting for over 70% of the patients in both disease cohorts.

Furthermore, the average hospital costs per patient (including out-of-pocket costs) and hospital utilization metrics (length of stay, LOS) were estimated. Among patients with depression, the median annualized total hospital cost per patient was $2,118.04 (in 2021 US dollars) for those with chronic pain, nearly double the cost for patients without pain ($1,174.82). A similar trend was observed in the functional limitation cohort: patients with pain incurred a median total cost of $3,211.39, compared to $2,619.88 for those without pain. Annualized out-of-pocket costs were also significantly higher for patients suffering from pain across both cohorts ($337.56 and $184.61 for depression patients; $337.55 and $163.40 for functional limitation patients).

Additionally, the annualized LOS per patient was consistently longer in the pain groups. Depression patients with chronic pain had a median LOS of 21.00 days (IQR: 11.50–36.50 days) compared to 11.00 days (IQR: 6.00–21.51 days) for those without pain. Among patients with functional limitations, the median stay was 26.50 days (IQR: 13.00–49.88 days) for the pain group and 20.50 days (IQR: 10.01–43.03 days) for the non-pain group. Notably, among the patients experiencing chronic pain, the vast majority reported pain in a single site (82.80% in the depression patients and 80.84% in the functional limitation patients).

### 3.2 Association analyses (step 1)

**Figure 1:**
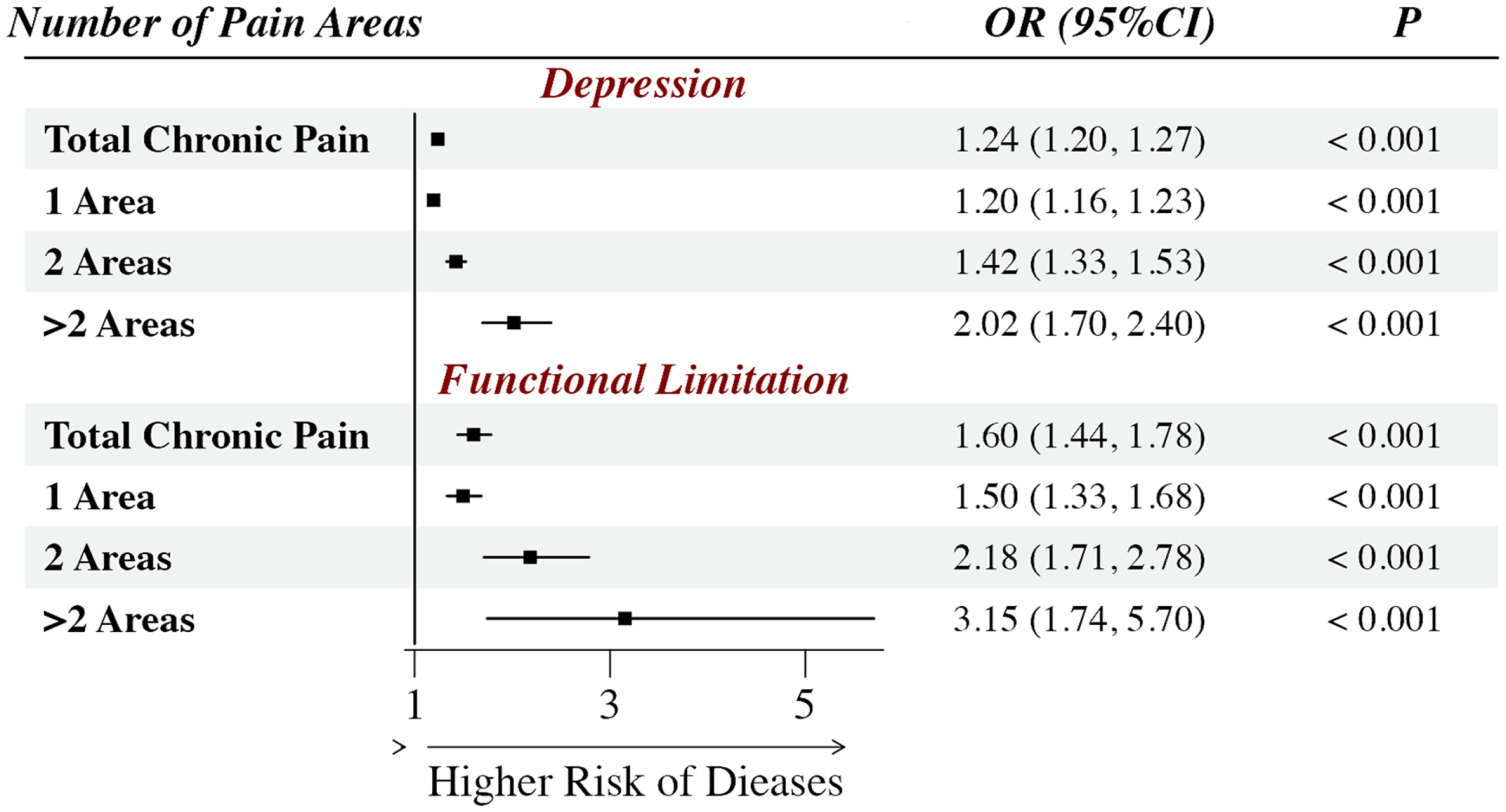
Association of chronic pain and hospitalization occurrence. OR is odds ratio; CI is confidence interval; the covariates have been controlled.

Figure 1 presents the results of the logistic regression, revealing a significant association between chronic pain and the odds of comorbid depression and functional limitation after adjusting for covariates. Compared to those without pain, the overall odds ratios (ORs) for depression and functional limitation were 1.24 (95% *CI*: 1.20–1.27) and 1.60 (95% *CI*: 1.44–1.78), respectively. Furthermore, stratified analyses demonstrated that these associations intensified with an increasing number of pain areas. For patients with depression, the odds nearly doubled for those with multi-site pain (> 2 areas; *OR* = 2.02, 95% *CI*: 1.70–2.40) compared to those with a single pain area (*OR* = 1.20, 95% *CI*: 1.16–1.23). Regarding functional limitation, this difference was even more pronounced: the odds were 3.15 times higher (*OR* = 3.15, 95% *CI*: 1.74–5.70) among individuals with > 2 pain areas compared to those without pain (all *p* < 0.001).

### 3.3 Attributable hospital costs (step 2)

To mitigate selection bias, Propensity Score Matching (PSM) successfully balanced baseline covariates between the chronic pain and control groups (Supplementary Figures S3, S4) prior to estimating separate hospital cost models for the depression and functional limitation cohorts.

**Figure 2:**
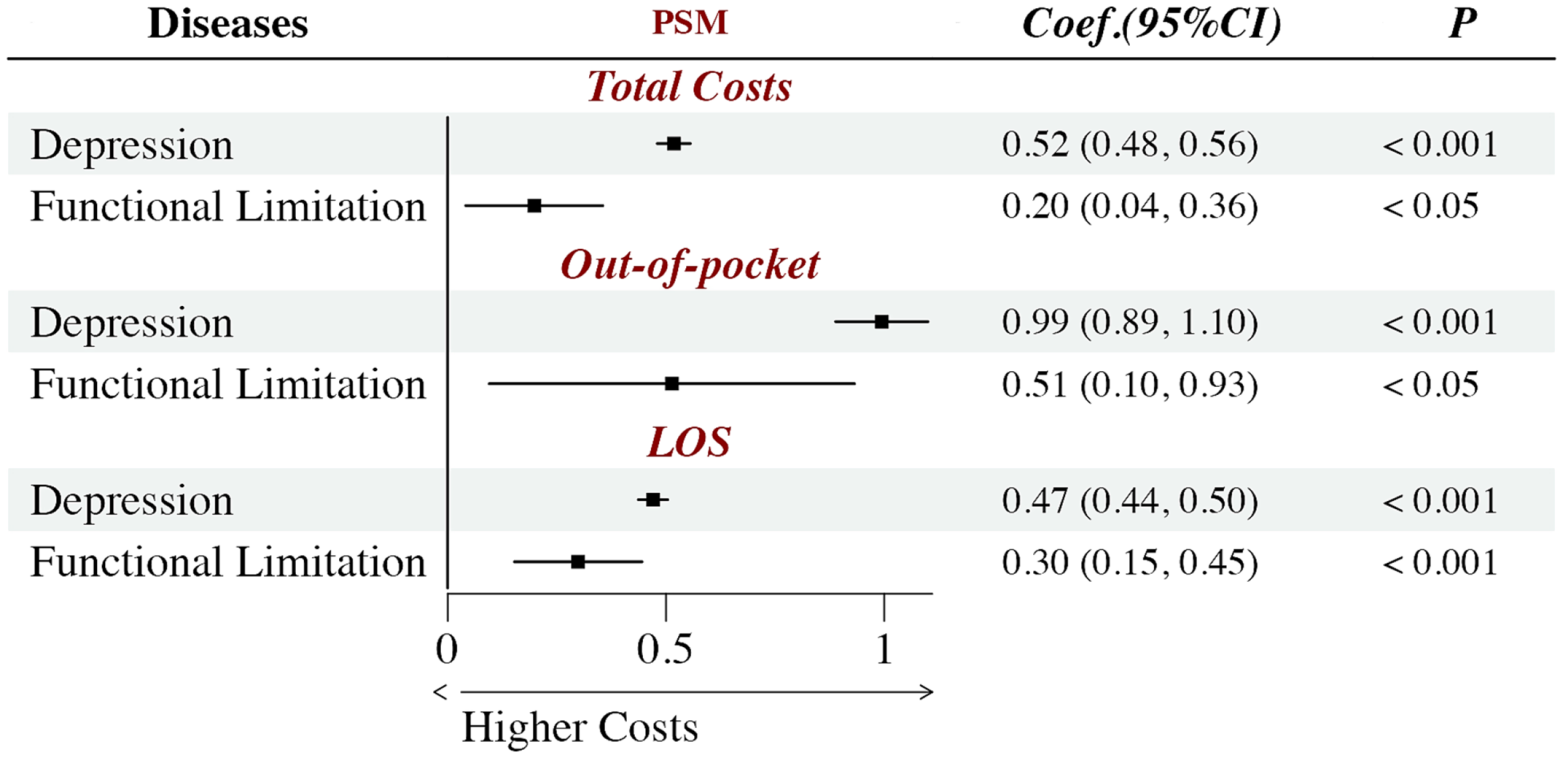
Association of chronic pain on hospital costs in depression and functional limitation patients. Total costs means the annually total hospital costs per patient; Out-of-pocket means the annually out-of-pocket costs per patient; LOS means the annually length of hospital stay per patient; Dependent variables (hospital costs) were natural log-transformed; Estimates are based on a propensity score matched (PSM) sample. Coef. is the regression coefficient; CI is confidence interval; The covariates have been controlled.

Figure 2 illustrates the associations between chronic pain and attributable hospital cost outcomes, after adjusting for all covariates. The presence of chronic pain was associated with significantly higher economic burdens across all evaluated metrics. Specifically, within the depression cohort, patients with chronic pain incurred 68.2% higher total hospital costs ( β =0.52, *p* < 0.001), 169.1% higher out-of-pocket costs ( β =0.99, *p* < 0.001) and a 60.0% longer LOS ( β =0.47, *p* < 0.001) compared to those without pain. Similarly, among patients with functional limitations, chronic pain also significantly increased total hospital costs ( β =0.20, *p* < 0.05), out-of-pocket costs ( β =0.51, *p* < 0.05), and LOS ( β =0.30, *p* < 0.001), which correspond to relative percentage increases of 22.1, 66.5, and 35.0%, respectively.

**Figure 3:**
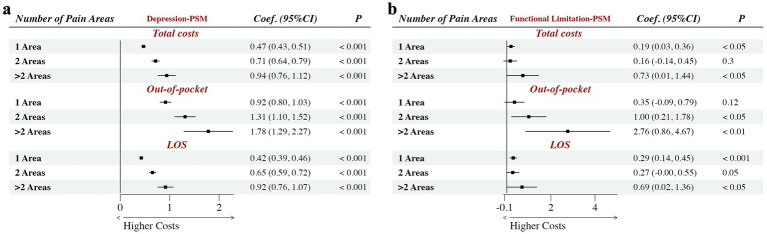
Association of multi-area pain on hospital costs in depression and functional limitation patients. **(a)** Is the result in depression patients; **(b)** is the result in functional limitation patients; total costs means the annually total hospital costs per patient; out-of-pocket means the annually out-of-pocket costs per patient; LOS means the annually length of hospital stay per patient; dependent variables (hospital costs) were natural log-transformed; estimates are based on a propensity score matched (PSM) sample. Coef. is the regression coefficient; CI is confidence interval; the covariates have been controlled.

As illustrated in Figure 3a, hospital costs among patients with depression were significantly associated with the number of reported pain areas. A clear gradient was observed, where costs escalated progressively with the accumulation of pain areas.

Compared to depression patients without pain, the presence of multi-area pain progressively exacerbated both clinical and economic burdens. For total hospital costs, expenses increased by 60.0% ( β =0.47, *p* < 0.001) for patients with a single pain area, escalating to a 156.0% increase ( β =0.94, *p* < 0.001) for those with more than two pain areas. A similar stepwise upward trend was observed for length of stay (LOS), showing relative increases of 52.2 and 150.9%, respectively. Most strikingly, out-of-pocket costs yielded the largest coefficient ( β = 1.78, *p* < 0.001) in the log-transformed model, which means the patients reporting more than two pain areas experienced 493.0% increase in out-of-pocket hospital costs.

As shown in Figure 3b, hospital costs among patients with functional limitations generally increased with the number of reported pain areas. Compared to patients without pain, total hospital costs and length of stay (LOS) significantly increased by 20.9 and 33.6% respectively, for those with a single pain area. These relative increases escalated to 107.5 and 99.4% for patients reporting with more than two pain areas (all *p* < 0.05). Furthermore, the presence of multi-area pain was associated with a rise in out-of-pocket costs, reaffirming the compounding economic burden of comorbid chronic pain.

### 3.4 Sensitivity analyses

Two sensitivity analyses were conducted to verify the robustness of our estimates. First, re-estimating the models using the unmatched data yielded consistent findings (Supplementary Figures S5, S6). Second, we performed regressions on the raw, unlogged variables to provide directly interpretable results on an absolute scale (Supplementary Figures S7, S8). Overall, these absolute estimates confirmed the attributable hospital costs of chronic pain within the depression patients, chronic pain significantly increased annualized total hospital costs by $1,108.82 and LOS by 11.37 days, (both *p* < 0.001). Similar robust increases in total hospital costs (by $924.31, *p* < 0.05) and LOS (by 24.98 days, *p* < 0.001) were observed in the functional limitation patients, although the absolute impact on out-of-pocket was not statistically significant (*p* = 0.37).

## 4 Discussion

We conducted a comprehensive analysis of the associations between chronic pain with depression and functional limitations among middle-aged and older adults. Additionally, we explored the attributable hospital costs associated with chronic pain within these two specific disease cohorts. Our findings unveiled three key insights: (1) the presence of chronic pain significantly increases the odds of comorbid depression and functional limitation; (2) chronic pain shows a positive association with increased hospital costs among patients with depression and functional limitation; (3) the presence of multi-area chronic pain further exacerbated the hospital costs for both groups.

### 4.1 Comparison to available studies

Most existing epidemiological studies rely on survey data to explore the relationship between pain and related diseases. However, these studies typically focus on a single outcome and frequently overlook the cumulative impact of multi-area pain. For instance, a national study in the United States showed presence of chronic pain in adults associated with higher possibility for depression (32). Similarly, research in Netherlands also demonstrated associations between presence of depressive disorders and symptom severity with different pain dimensions, intensity, and the location (33); a telephone survey of in the United Kingdom, Germany, Italy, Portugal, and Spain also provided the evidence (34). Regarding functional capacity, several studies employing activities of daily living (ADLs) as an assessment tool have found significant correlations between pain and functional decline, revealing strong cross-sectional associations (24, 25). Despite this body of evidence, inconsistencies in the literature remain. In the Chinese context, only a few population-based studies have provided empirical evidence linking chronic pain to depression (35) and diminished functional capacity (36).

However, traditional population-based surveys inherently lack precision in quantifying hospital costs. To better understand and mitigate the clinical and economic burdens of chronic pain, quantifying its direct impact on the hospital costs of comorbid conditions is a critical step. Currently, literature addressing this specific issue remains sparse. By utilizing precise cost calculations derived from comprehensive hospital discharge data, this study addresses this critical research gap. Ultimately, our findings underscore that prioritizing the health management of older adults with chronic pain can yield substantial clinical and economic dividends. Implementing proactive pain interventions represents a highly cost-effective strategy to curb escalating hospital costs, particularly when targeted at the highly vulnerable demographic suffering from multi-area pain.

### 4.2 Clinical and policy implications

This study emphasizes the importance of paying greater attention to the emotional well-being and chronic complication of patients suffering from chronic pain, particularly multi-area pains. Chronic pain has a high incidence worldwide, the prevalence of chronic pain among adults in US was 20.9% (37), 43.5% in UK (38), 18.9% in Canada (39), and the overall pooled prevalence was about 18% in developing countries (40). Complete elimination of pain is seldom achievable for an extended period. As a result, it is crucial for patients and clinicians to establish treatment goals that prioritize pain reduction, functional improvement, and enhanced quality of life. Optimal outcomes in chronic pain management are more likely when co-occurring mental health conditions, such as depression, are effectively addressed (41). To reduce health risk, health care providers and researchers should consider regularly screening for depression, and functional limitations. In general, healthcare professionals, researchers and policymaker are encouraged to adopt a holistic approach that integrates psychological support into the treatment and management of chronic pain, ensuring both the physical and emotional needs of patients are adequately addressed.

Hospitalization caused by depression has been indicated as one of Ambulatory Care Sensitive Conditions (ACSCs), which be identified as those hospitalizations could be avoidable by timely and effective primary and out-hospital care (42). However, our results indicate that the presence of chronic pain may complicate this prevention situation and become one of the important factors contributing to the increase in medical resource usage of older adults. The results of this study show that patients with depression with pain have significantly higher total costs and hospitalizations than those without pain. This economic burden indicates a key defect in the current health service system: the disconnection between physical chronic pain management and mental health services. Therefore, in order to reduce the pressure on health system, health management strategies could be advised to shift to proactive and community-based approaches. It is necessary to ensure that the health system strengthens the out-of-hospital management of chronic pain, particularly focusing on the maintenance of mental health and policy attention for individuals with multiple pain areas. By integrating mental health screening and psychosocial interventions into routine pain management, healthcare providers can break the “vicious cycle” between pain and depression, thereby reducing the number of preventable hospitalizations and hospitalization costs, and improving overall cost-effectiveness (43).

## 5 Strength and limitations

This study has several strengths. First, we utilized individual-level inpatient data, which provides comprehensive details regarding hospitalization characteristics and hospital costs. Second, to the best of our knowledge, this is the first study to specifically investigate how chronic pain drives the escalation of hospital costs for associated conditions. This finding offers valuable insights for outpatient care and comorbidity management. Third, our research highlights the critical clinical and economic impact of multi-area chronic pain, thereby providing robust empirical evidence for future studies in this domain.

However, this study has several limitations. First, certain unobserved confounders may persist beyond the scope of control, posing challenges in inferring causal effects. Second, while this study did not employee the impact of specific pain areas, the relationship between these areas may be complex and merits further investigation in future studies. Third, the data is cross-sectional and prevented us to get the all the status of pain intensity, more follow-up analysis with cohort study design is expected to be constructed for the dose response association (33). Furthermore, because the study data are derived exclusively from hospitalization records, admission rate bias (Berkson’s paradox) may exist. Consequently, our sample may over represent individuals with more severe clinical profiles, and the findings may not be fully generalizable to patients managed in outpatient or community-based settings. Moreover, patients suffering from both chronic pain and comorbidities (such as depression or functional limitation) inherently have a higher probability of being admitted to the hospital than those with a single condition. As a result, the associations and the calculated attributable hospital costs observed in this hospitalized cohort might be overestimated compared to the general, non-hospitalized population.

## 6 Conclusion

This analysis adds to a growing body of literature documenting the associations of chronic pain with depression and functional limitations by utilizing detailed inpatient data. Furthermore, to the best of our knowledge, this study may be the first to explicitly examine attributable hospital costs associated with chronic pain in older adults. These findings provide actionable insights, guiding clinicians and health policy makers toward targeted priorities and interventions. Our findings also emphasize that, alongside preserving physical function, the importance of maintaining the mental health of patients with chronic pain is crucial to avoid more unnecessary and avoidable hospital costs. Notably, our findings suggest that patients with multi-site chronic pain constitute a highly vulnerable population. This underscores an urgent need to prioritize comprehensive pain management within emergency and outpatient care settings, thereby mitigating preventable hospital costs among middle-aged and older adults.

## Data Availability

The data analyzed in this study is subject to the following licenses/restrictions: The Inpatient Discharge Dataset of Sichuan Province sourced from the Health Information Center of Sichuan Province. The use of the data requires approval from the Health Commission of Sichuan Province. Requests to access these datasets should be directed to Health Commission of Sichuan Province (https://wsjkw.sc.gov.cn/scwsjkw/index.shtml).
