# Supplementary material for: Association and attributable hospital costs of chronic pain with relevant geriatric sensitive diseases among the older adults
Source: Front Public Health. 2026 May 1;14:1828836. doi: 10.3389/fpubh.2026.1828836 (PMC13147608; doi:10.3389/fpubh.2026.1828836)
Supplement: Supplementary file 1 [file Data_Sheet_1.docx]

**Supplementary file**

# Association and Attributable Hospital Costs of chronic pain with relevant geriatric sensitive diseases among the older adults

**Ting Chen, Tianjiao Lan, Kun Tan, Jay Pan, Xiuli Wang**

**Contents**

[Text S1 Details of covariates 2](#_Toc227072931)

[Figure S1 The diagram of data cleaning 4](#_Toc227072932)

[Figure S2 Residual diagnostic plots for regression models using untransformed outcomes 5](#_Toc227072933)

[Figure S3 Detailed diagnostics and matching performance of PSM in Depression patients 6](#_Toc227072934)

[Figure S4 Detailed diagnostics and matching performance of PSM in Functional limitation patients 7](#_Toc227072935)

[Figure S5 Association of Chronic Pain on Hospital Costs in Depression and Functional Limitation Patients 8](#_Toc227072936)

[Figure S6 Association of Muti-area Pain on Hospital Costs in Depression and Functional Limitation Patients 9](#_Toc227072937)

[Figure S7 Association of Chronic Pain on Hospital Costs in Depression and Functional Limitation Patients 10](#_Toc227072938)

[Figure S8 Association of Muti-area Pain on Hospital Costs in Depression and Functional Limitation Patients 11](#_Toc227072939)

[Table S1 ICD-10 for chronic pain and outcomes in Inpatient Discharge Dataset 12](#_Toc227072940)

[Table S2 The distribution of pain areas in Inpatient Discharge Dataset 14](#_Toc227072941)

**Text S1 Details of covariates**

In this paper, in order to obtain stable results, we control for a range of participant characteristics, specifically including:

(1) *age.* Recorded the participant’s age in 2021 as a continuous variable with a minimum unit of 1 year.

(2) *sex.* Recorded the gender of the participant as a categorical variable. It is divided into male and female.

(3) *ethnic group*. Recorded the ethnic group of the participant as a categorical variable. It is divided into minority (the Han nationality) and non-minority.

(4) *Marital status*. Recorded the marital status of the participant as a categorical variable. It is divided into “Married” and “Unmarried, divorced or widowed”.

(5) *Occupation type*. Recorded the Occupation type of the participant as a categorical variable. It is divided into “Mental worker” (including Civil Servants, Professional and Technical Personnel, Office Staff/Clerical Workers, Corporate Management Personnel, Students), “Manual workers” (including Manual Workers/Industrial and Workers and Farmers/Agricultural Workers) and “Uncertain” (other occupations or occupational statuses).

(6) *Health insurance*. Recorded whether participate in medical insurance of the participant as a categorical variable. The medical insurance includes Employee medical insurance, Resident medical insurance and Others (such as having no insurance or only commercial medical insurance).

(8) *Regional economic level*. The economic development level of the patient's permanent residence county (using the per capita GDP of the county in 2021 as the indicator) is divided into three levels: high, medium, and low, and it is a categorical variable.

**
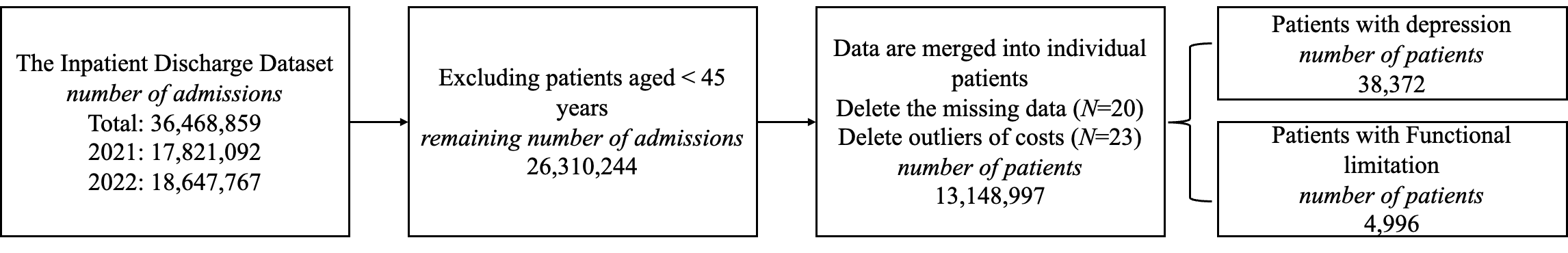
**

**Figure S1 The diagram of data cleaning**

**
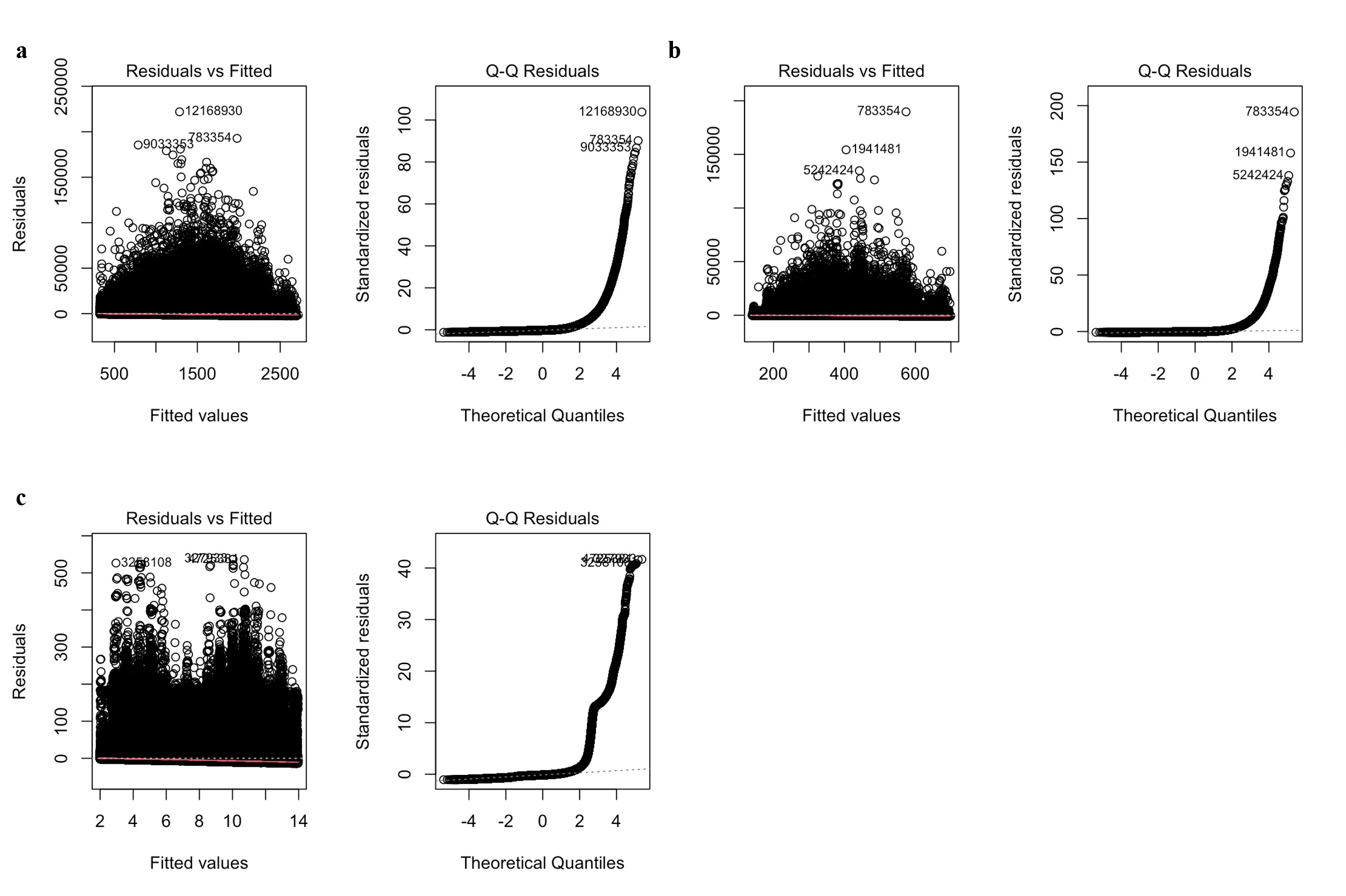
**

**Figure S2 Residual diagnostic plots for regression models using untransformed outcomes**

Notes: The panels display the residuals versus fitted values (left) and Normal Q-Q plots of standardized residuals (right) for models predicting: (**a**) total hospital costs, (**b**) out-of-pocket costs, and (**c**) length of stay (LOS). The pronounced funnel shape in the residual plots and the severe upward deviation in the right tails of the Q-Q plots indicate substantial heteroscedasticity and right-skewness when relying on unlogged outcome variables.

**
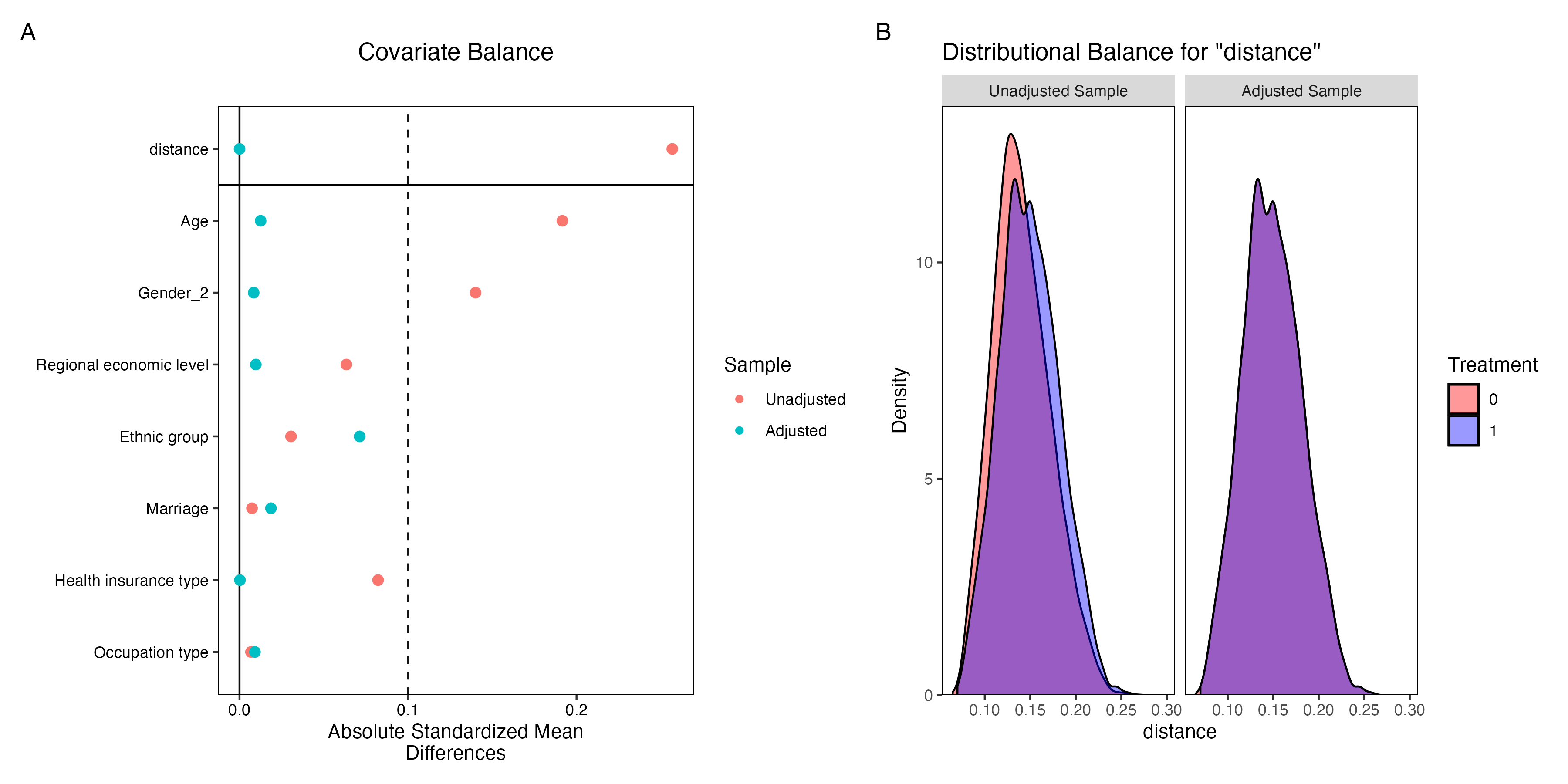
**

**Figure S3** **Detailed diagnostics and matching performance of PSM in Depression patients**

**
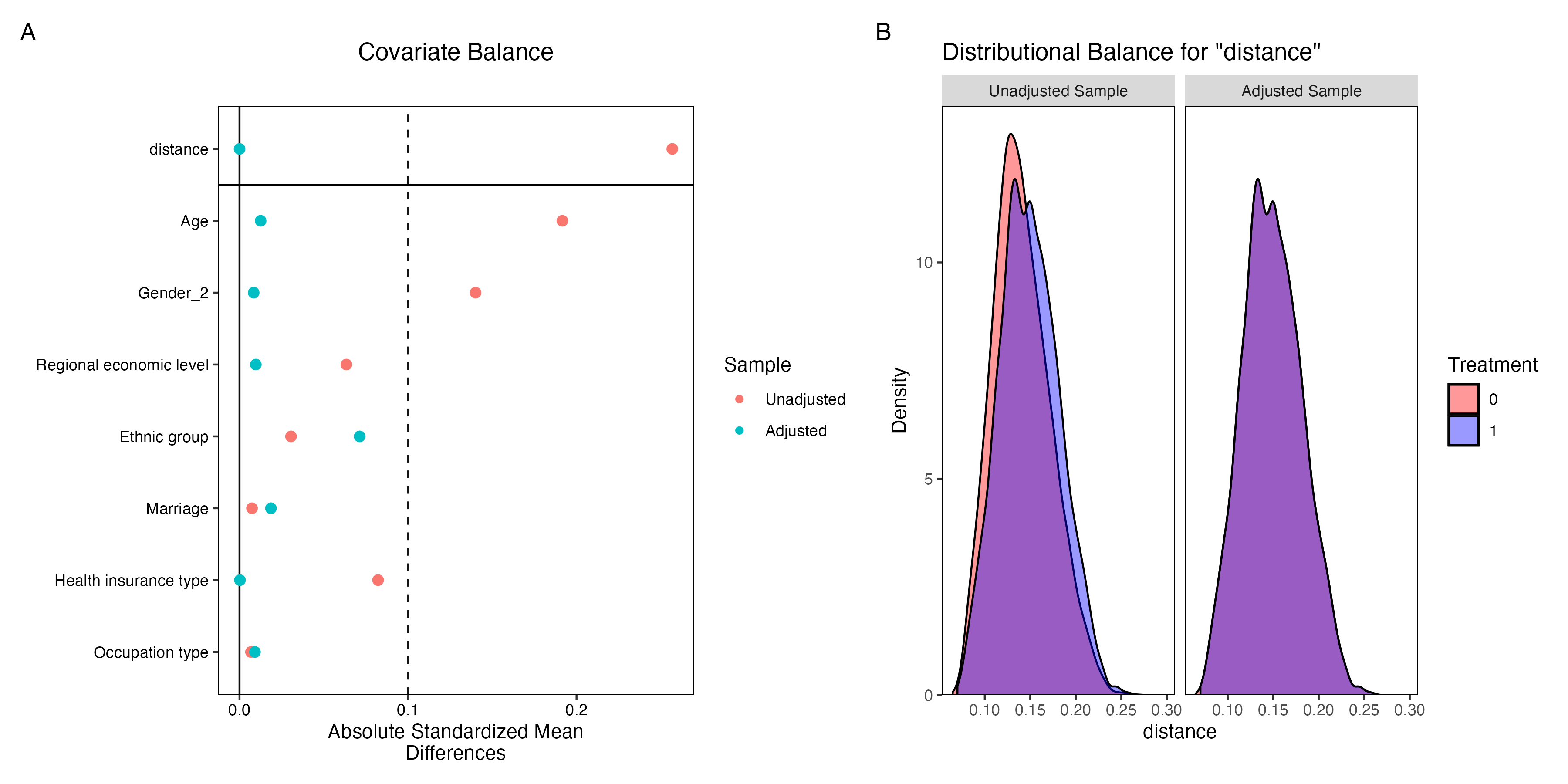
**

**Figure S4 Detailed diagnostics and matching performance of PSM in Functional limitation patients**

**
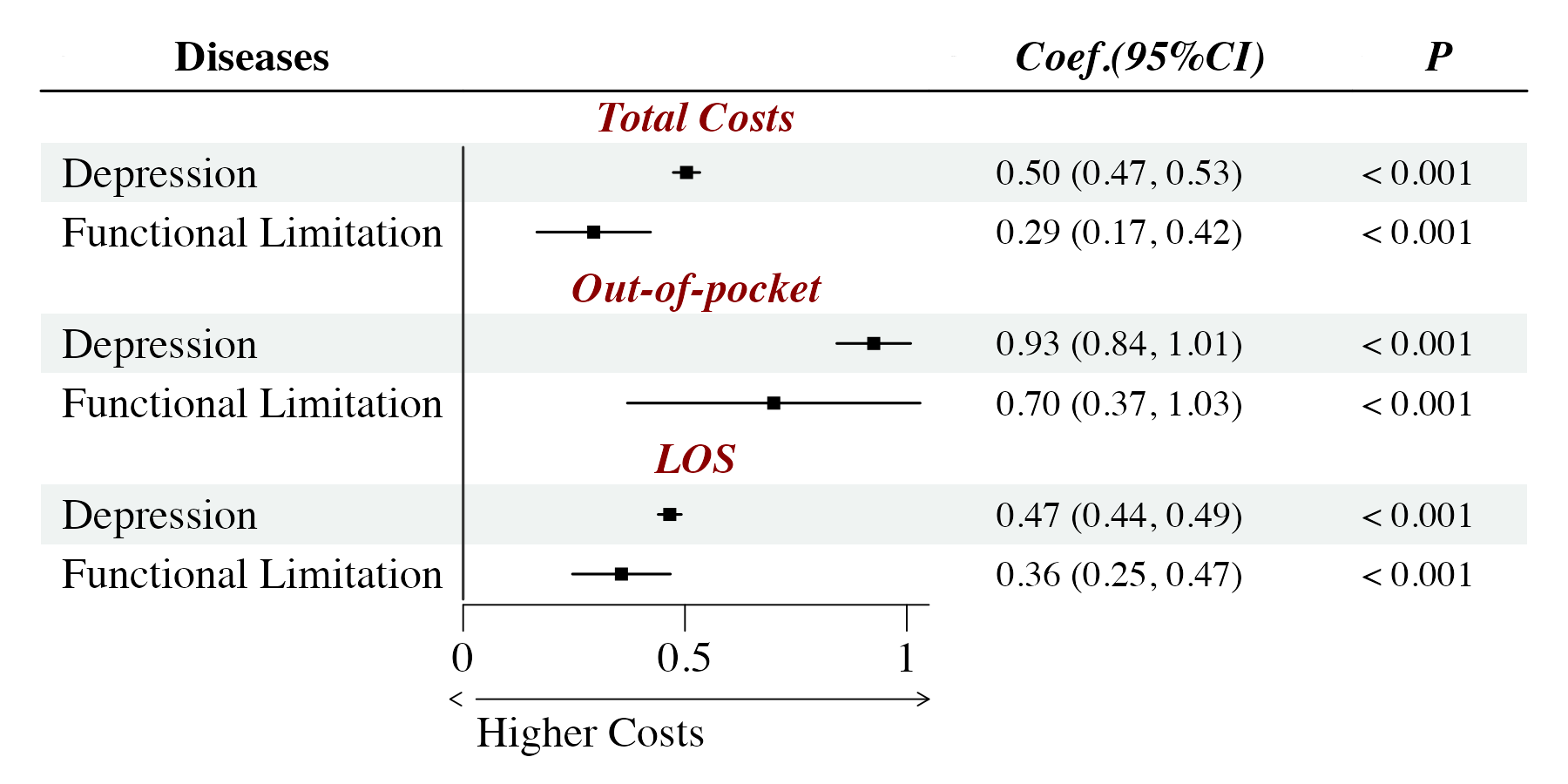
**

**Figure S5 Association of Chronic Pain on Hospital Costs in Depression** **and Functional Limitation Patients**

Notes: Total costs means the annually total hospital costs per patient; Out-of-pocket means the annually Out-of-pocket costs per patient; LOS means the annually length of hospital stay per patient; Dependent variables (hospital costs) were natural log-transformed; *Coef.* is the regression coefficient; *CI* is confidence interval; The covariates have been controlled.

**
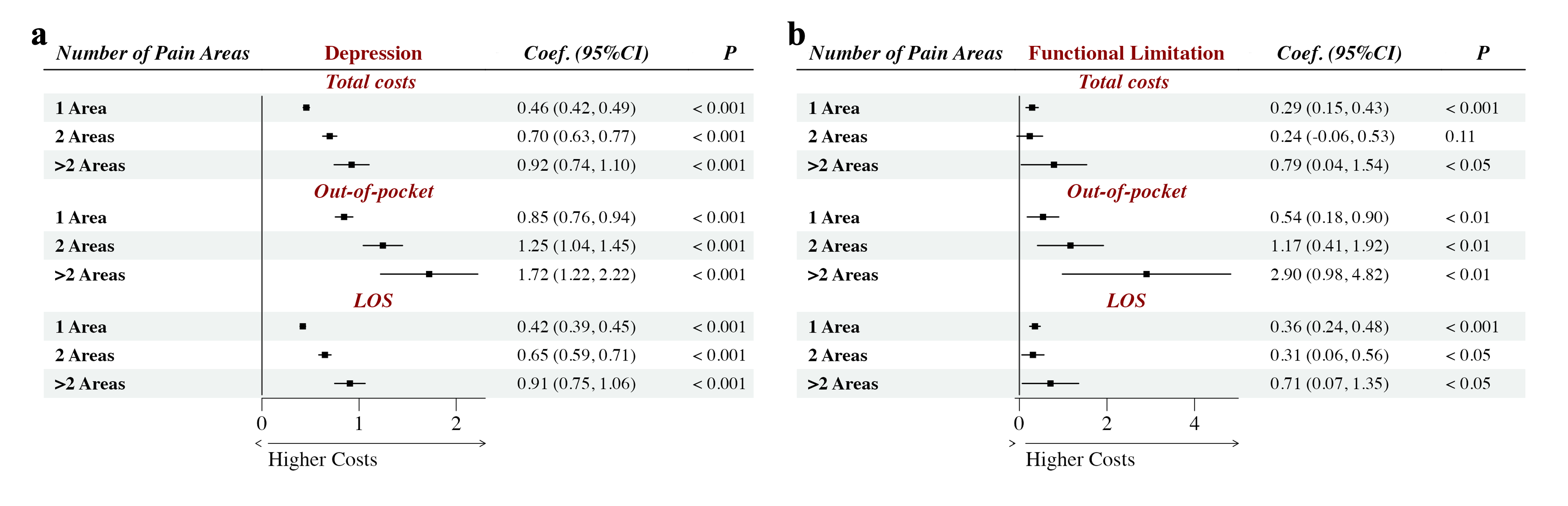
**

**Figure S6 Association of Muti-area Pain on Hospital Costs in Depression and Functional Limitation Patients**

Notes: (a) is the result in Depression patients; (b) is the result in Functional Limitation Patients; Total costs means the annually total hospital costs per patient; Out-of-pocket means the annually Out-of-pocket costs per patient; LOS means the annually length of hospital stay per patient; Dependent variables (hospital costs) were natural log-transformed; *Coef.* is the regression coefficient; *CI* is confidence interval; The covariates have been controlled.

**
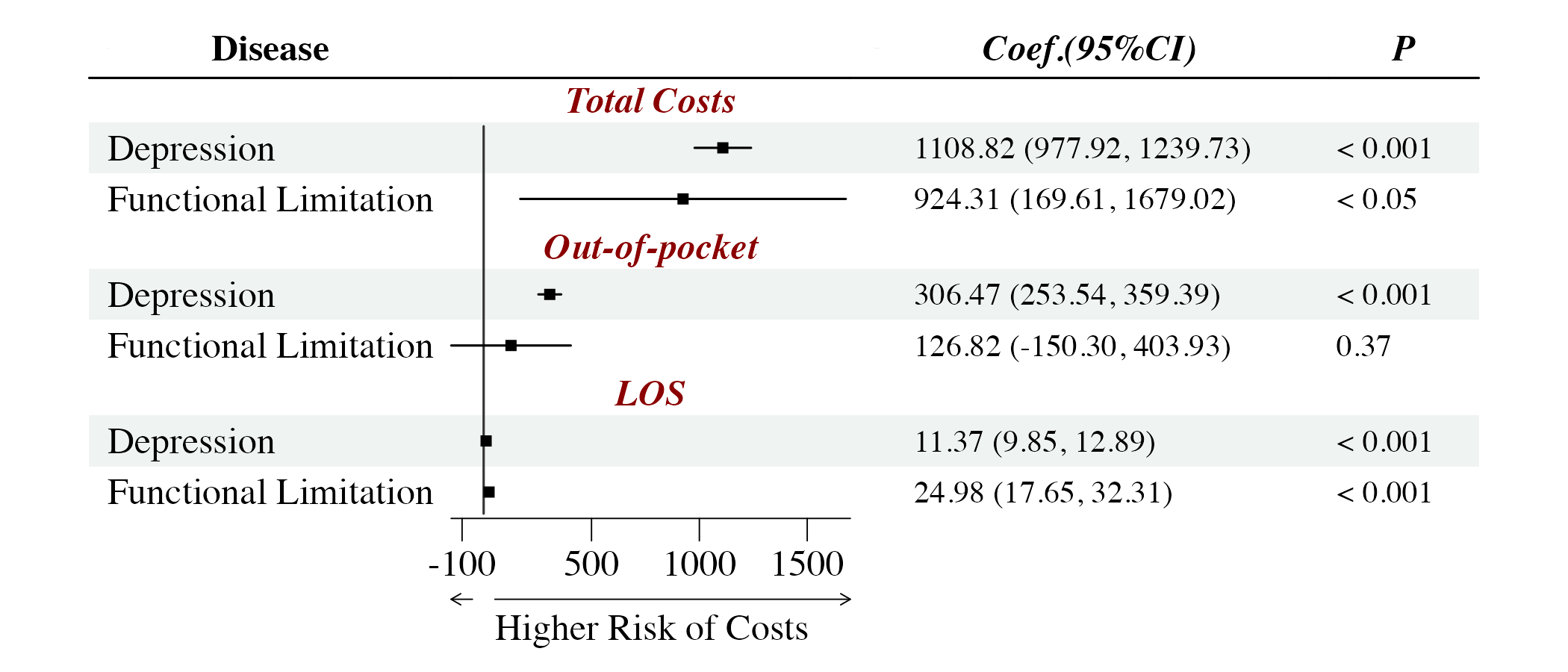
**

**Figure S7 Association of Chronic Pain on Hospital Costs in Depression and Functional Limitation Patients**

Notes: Total costs means the annually total hospital costs per patient; Out-of-pocket means the annually Out-of-pocket costs per patient; LOS means the annually length of hospital stay per patient; *Coef.* is the regression coefficient; *CI* is confidence interval; The covariates have been controlled.

**
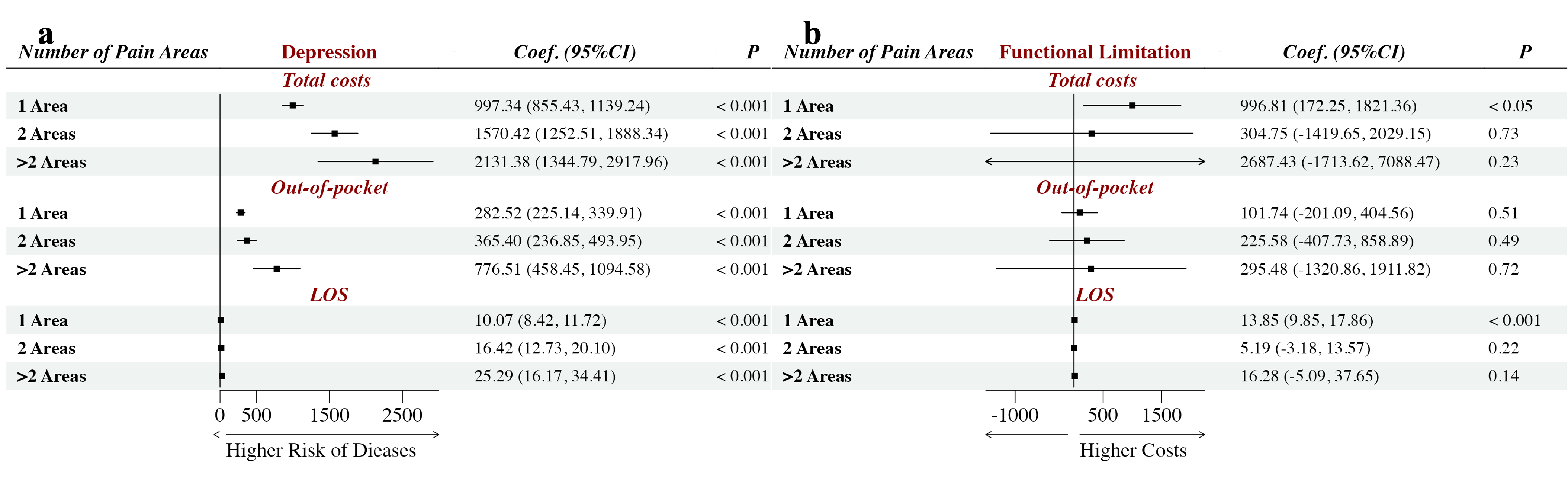
**

**Figure S8 Association of Muti-area Pain on Hospital Costs in Depression and Functional Limitation Patients**

Notes: (a) is the result in Depression patients; (b) is the result in Functional Limitation Patients; Total costs means the annually total hospital costs per patient; Out-of-pocket means the annually Out-of-pocket costs per patient; LOS means the annually length of hospital stay per patient; *Coef.* is the regression coefficient; *CI* is confidence interval; The covariates have been controlled.

**Table S1 ICD-10 for chronic pain and outcomes** **in Inpatient Discharge Dataset**

| **Sites or Outcomes** | **Principal chronic pain^a^** | **Chronic secondary pain syndromes^b^** |
| --- | --- | --- |
| ***Head*** | *G44.2 Tension type headache G43.1 Migraine G50.1 Atypical facial pain* | *G44.4 Drug-induced headache* |
| ***Shoulder*** | *M79.600x011 Pain in shoulder M25.51 Non-specific shoulder pain* | *M19.019 Osteoarthritis acromioclavicular joint  M75.4 Impingement syndrome of shoulder  M75.1 Rotator cuff syndrome  S43.0 Dislocation of shoulder joint  M75.0 Adhesive capsulitis of shoulder* |
| ***Arm*** | *M79.600x021 M79.600x02 Pian in arm M25.52 Pain in elbow* | *M19.0 Osteoarthritis of elbow joint M77 Epicondylitis  S46.1 Injury long head of biceps tendon M75.2 Bicipital tendinitis* |
| ***Hand and Finger*** | *M79.601 Pain in hand and fingers* | *M19.04/18.0 Osteoarthritis of hand/fingers G56.2 Lesion of ulnar nerve  G56.0 Carpal tunnel syndrome* |
| ***Chest*** | *R07.4 Unspecified chest pain* |  |
| ***Stomach and Abdominal pain*** | *R10.4 Unspecified abdominal pain K58 Irritable Bowel Syndrome* | *K45.0 Abdominal hernia K51.2 Ulcerative proctitis  K62.7 Radiation proctitis* |
| ***Back*** | *M54.6 Pain in thoracic spine* | *S22.0 Fracture thoracic  M80.5 Osteoporosis* |
| ***Waist*** | *M54.5 Low back pain* | *M54.4 Lumbago with sciatica  G54.0 Brachial plexus disorders* |
| ***Buttock*** | *M25.55 Pain in hip* | *M16 Osteoarthritis of hip* |
| ***Leg*** | *M79.600x051 M79.600x05 Pain in leg* | *M23.3 Other meniscus derangements  M70.6 Trochanteric bursitis  S72.23Displaced fracture of femur* |
| ***Knee*** | *M25.56 Pain in knee M79.665 Knee Pain syndrome* | *M17 Osteoarthritis of knee* |
| ***Foot, Ankle and Toes*** | *M79.603 Pain in toes M79.604 Pain in foot*  *M79.602 Pain in heel M79.600x002 Tarsalgia* | *M19.0 Osteoarthritis of Foot  M72.2 Plantar Fasciitis  M20.1 Hallux valgus  L84 Corns  M76.6 Achilles tendinitis  G57.6 Morton’s metatarsalgia*  *M24.27Disorder of ligament, ankle* |
| ***Neck*** | *M54.2 Cervicalgia M53.1 Cervicobrachial syndrome* | *M54.12 Cervical radiculopathy* |
| ***Outcomes*** |  | |
| ***Depression*** | *F32 F33* | |
| ***Abilities Decline in basic or physical activities of daily living*** | *R26 Abnormal gait and movement* *R29.3 Dyspnea on exertion*  *Z73 Limitation of activities due to disability* | |

**^a^**: ICD-10 pain symptom codes of the principal chronic pain condition.

^b^: ICD-10 pain symptom codes classified as chronic secondary pain syndromes.

**Table S2 The distribution of pain areas in Inpatient Discharge Dataset**

| **Areas** | ***Total individual Number*=** **26,297,994** | |
| --- | --- | --- |
|  | **Depression** | **Functional Limitation** |
|  | **(*N*= 38,372)** | **(*N*=** **4,996)** |
| ***Head*** |  |  |
| No | 37,983(98.99) **^a^** | 4,974 (99.56) |
| Yes | 389 (1.01) | 22 (0.44) |
| ***Shoulder*** |  |  |
| No | 37,606 (98.00) | 4,792 (95.92) |
| Yes | 3,83 (2.00) | 204 (4.08) |
| ***Arm*** |  |  |
| No | 38,235 (99.64) | 4,970 (99.48) |
| Yes | 137 (0.36) | 26 (0.52) |
| ***Hand and Finger*** | |  |
| No | 38,323 (99.87) | 4,988 (99.84) |
| Yes | 49 (0.13) | 8 (0.16) |
| ***Chest*** | |  |
| No | 38,214 (99.59) | 4,980 (99.68) |
| Yes | 158 (0.41) | 16 (0.32) |
| ***Stomach and Abdominal pain*** | |  |
| No | 37,293 (97.19) | 4,932 (98.72) |
| Yes | 1,079 (2.81) | 64 (1.28) |
| ***Back*** | |  |
| No | 37,909 (98.79) | 4,924 (98.56) |
| Yes | 463 (1.21) | 72 (1.44) |
| ***Waist*** |  |  |
| No | 37,646 (98.11) | 4,896 (98.00) |
| Yes | 726 (1.89) | 100 (2.00) |
| ***Buttock*** | |  |
| No | 38,122 (99.35) | 4,934 (98.76) |
| Yes | 250 (0.65) | 62 (1.24) |
| ***Leg*** | |  |
| No | 38,008 (99.05) | 4,924 (98.56) |
| Yes | 364 (0.95) | 72 (1.44) |
| ***Knee*** | |  |
| No | 36,378 (94.80) | 4,664 (93.35) |
| Yes | 1,994 (5.20) | 332 (6.65) |
| ***Foot, Ankle and Toes*** | |  |
| No | 38,306 (99.83) | 4,974 (99.56) |
| Yes | 66 (0.17) | 22 (0.44) |
| ***Neck*** |  |  |
| No | 38,259 (99.71) | 4,982 (99.72) |
| Yes | 113 (0.29) | 14 (0.28) |

**^a^**: discrete variables are presented as n (%)
